# Puerperal Convulsions from Gastric Irritation

**Published:** 1858-04

**Authors:** J. Levergood

**Affiliations:** Wrightsville, Pa.


### *Puerperal Convulsions from Gastric Irritation.* By J. LEVERGOOD, M. D., Wrightsville, Pa.

Notwithstanding gastric irritation is conceded to be a not infrequent cause of eclampsia, the subjoined history of a case of this sort, which occurred in my practice, may possibly present some features of sufficient practical importance to render it worthy of publication.

October 31st, 1853, I was requested, about five o'clock, P. M., to call to see Mrs. V., and knowing her to be near the end of her pregnancy, I visited her as speedily as possible, supposing she had been taken in labor. Upon my arrival I found her walking up and down the room complaining greatly of a pain in the pit of the stomach, but in other respects enjoying her usual good health. The seat of the pain from which she was suffering, its continuous and unintermitting character, in connection with the undilated condition of the os uteri, led me to conclude that she was not in the incipient stage of parturition. After repeatedly interrogating her, I at length ascertained that she had eaten a piece of *raw turnip;* but the portion she had swallowed was so very small, as in her opinion, not to deserve mentioning, in fact not exceeding, as she expressed it, *a bite.* Although an emetic seemed clearly indicated, I felt averse to administering it, apprehensive that by its powerful excito-motory action premature labor might be broght about, and partly, also, because the patient herself was strongly opposed, under the circumstances, to the use of such a remedy. I therefore ordered a large dose of castor oil, with forty drops of elixir of opium, with instructions to repeat the elixir in an hour, provided there was no mitigation of the pain. At eleven o'clock I was sent for in great haste, the messenger informing me that the patient had been suddenly attacked with convulsions. Finding her upon my arrival with a livid countenance, distorted features, frothing at the mouth, agitation of the body, and coma, I at once opened a vein in the arm, and abstracted a large amount of blood. An examination per vaginum also revealed the first stage of labor nearly completed, the vertex presenting. With the concurrence of Dr. Barton Evans, of whose valuable assistance I had the benefit, I determined to let the labor take its natural course, upon the ground that "a meddlesome midwifery is bad," and "that more women die when they are officiously delivered by force' than when they are committed to their own resources." The result verified the wisdom of the course we adopted, for shortly afterward, and synchronously with a powerful spasm, the uterus contracted and expelled a still-born male child. The convulsions continued for two days, and a slow recovery then took place, a most rigid antiphlogistic treatment having been employed.

That the piece of turnip was the exciting cause of the convulsions seems very evident from the fact that, in seven prĕvious labors, and three subsequent to the above-mentioned attack, no untoward symptom occurred. That matters of an indigestible nature, taken into the stomach of a parturient female af a nervous temperament, will cause eclampsia, is an established fact in obstetric medicine, but the *cur et quere* have never been satisfactorily explained.—*N. A. Medico-Chirurgical Review.*

